# Validation of administrative claims to identify ultrasound enhancing agent use

**DOI:** 10.1186/s44156-023-00038-5

**Published:** 2024-02-07

**Authors:** Jordan B. Strom, Yang Song, Wenting Jiang, Yingbo Lou, Daniel N. Pfeffer, Omnya E. Massad, Pierantonio Russo

**Affiliations:** 1 Department of Medicine, Cardiovascular Division, Beth Israel Deaconess Medical Center, 375 Longwood Avenue, 4th floor, Boston, MA 02215 USA; 2 Richard A. and Susan F. Smith Center for Outcomes Research in Cardiology, Beth Israel Deaconess Medical Center, Boston, MA USA; 3 Harvard Medical School, Boston, MA USA; 4 EVERSANA®, LLC, Milwaukee, WI USA

**Keywords:** Ultrasound enhancing agents, Echocardiography, Administrative claims, Validation

## Abstract

**Background:**

Ultrasound enhancing agents (UEAs) are an invaluable adjunct to stress and transthoracic echocardiography (STE) to improve left ventricular visualization. Despite multiple single center studies evaluating UEA use, investigation into the rates, sources of variation, and outcomes of UEA use on a national level in the United States (US) has been limited by lack of validation of UEA codes for claims analyses.

**Methods:**

We conducted a retrospective cross-sectional study, 2019–2022, using linked multicenter electronic medical record (EMR) data from > 30 health systems linked to all-payor claims data representing > 90% of the US population. Individuals receiving STE in both EMR and claims data on the same day during the study window were included. UEA receipt as identified by presence of a Current Procedural Terminology (CPT) or National Drug Code (NDC) for UEA use within 1-day of the index STE event. We evaluated the performance of claims to identify UEA use, using EMR data as the gold standard, stratified by inpatient and outpatient status.

**Results:**

Amongst 54,525 individuals receiving STE in both EMR and claims data, 12,853 (23.6%) had a UEA claim in EMR, 10,461 (19.2%) had a UEA claim in claims, and 9140 (16.8%) had a UEA claim in both within the 1-day window. The sensitivity, specificity, accuracy, positive, and negative predictive values for UEA claims were 71.1%, 96.8%, 90.8%, 87.4%. and 91.6% respectively. However, amongst inpatients, the sensitivity of UEA claims was substantially lower (6.8%) compared to outpatients (79.7%).

**Conclusions:**

While the overall accuracy of claims to identify UEA use was high, there was substantial under-capture of UEA use by claims amongst inpatients. These results call into question published rates of UEA use amongst inpatients in studies using administrative claims, and highlight ongoing need to improve inpatient coding for UEA use.

**Supplementary Information:**

The online version contains supplementary material available at 10.1186/s44156-023-00038-5.

## Introduction

Ultrasound enhancing agents (UEAs) are an indispensable and underutilized tool to improve left ventricular opacification on stress or transthoracic echocardiography (STE) [1]. Consisting of a high-molecular weight gas surrounded by a phospholipid shell, UEAs provide effective endocardial border resolution and have been shown to demonstrate benefit in a variety of clinical circumstances including identification of wall motion abnormalities or apical pathology including apical aneurysms, characterization of the vascularity of cardiac tumors, and assessment of myocardial perfusion [1, 2]. Use of UEAs may additionally improve agreement between left ventricular volumes and ejection fraction on STE and cardiac magnetic resonance imaging and may improve reader confidence in excluding left atrial appendage thrombus on transesophageal echocardiography prior to cardioversion [1, 2]. While single center studies have suggested UEAs are associated with significant reductions in downstream cost, perhaps through avoidance of downstream testing and procedures performed due to diagnostic uncertainty [3, 4], multicenter datasets representative of the entire spectrum of clinical practice are needed to confirm that these benefits are widespread.

National databases of administrative billing claims represent attractive data sources to study UEA use due to the routine use of claims in the provision of clinical care as well as the presence of unique claims for UEA usage. However, the use of billing claims to study outcomes associated with UEA receipt has been limited to date, in part due to the uncertainty about the validity of these claims to accurately capture UEA administration. As secondary use of billing claims to identify cardiac procedures and health outcomes of interest has been shown to be valid in certain circumstances [5] but not others [6, 7], it is important to validate that claims for UEA use reasonably identify individuals who received UEAs in practice. Without this validation, it is challenging to identify, on a national scale, whether UEAs are underutilized, the clinical and hospital factors associated with decreased appropriate use, or whether UEAs are associated with improved short and long-term outcomes.

Accordingly, we aimed to evaluate the validity of UEA and STE codes in a large database of electronic medical record (EMR) records linked to all-payor claims in the US and assess how the validity differs by inpatient vs. outpatient status. We hypothesized that UEA claims would validly identify individuals who received UEAs in the EMR records, regardless of inpatient or outpatient status.

## Methods

### Study population

Individuals in the EVERSANA® Integrated EMR Database (EIED), 2019–2022, were considered for inclusion. The EIED is an aggregation and standardization of EMR data from over 50 unique EMRs (e.g., Allscripts®, CERNER®, GE®, Varian®, NextGen®, etc.) covering > 160 million US patients, including information from > 2000 physician offices and ambulatory health centers, and > 500 hospitals across more than 30 health systems. EMR data in the EIED has previously been directly linked to the Open Claims dataset represents an aggregation of all-payor administrative diagnosis, procedure, and pharmacy claims from 300 million US individuals, encompassing nearly 90% of the US population, with demographic distributions matching those of the overall US. The study was approved by the Institutional Review Board at Beth Israel Deaconess Medical Center, Boston, US with a waiver of informed consent.

### Exposures and outcomes

Receipt of STE was defined using standard Current Procedural Terminology (CPT) codes (Additional file 1: Table S1) [8]. Receipt of UEAs was defined as presence of a CPT or National Drug Code (NDC) for UEAs (Additional file 1: Table S2) within 1 day of the index STE, to allow for a lag in billing. Only individuals with STE in claims and EMR on the same day were included. Receipt of STE or UEAs respectively in the linked EMR data was used as the gold-standard for receipt.

### Statistical methods

Characteristics of individuals included are presented using numbers and proportions and compared between groups (i.e., identification of UEAs in claims vs. identification of UEAs in EMR data) using Pearson Chi-squared tests. The aggregated rates of STE or UEA receipt as ascertained via claims were compared to receipt of TTEs or UEAs in the linked EMR data to calculate the sensitivity, specificity, accuracy, positive (PPV) and negative (NPV) predictive value of each claims algorithm. These analyses were subsequently repeated, stratified by a patient’s inpatient or outpatient status. All analyses were performed using JMP Pro v17 (SAS Institute, Cary, NC) using a two-tailed p-value < 0.05 for significance.

## Results

### Overall results

Amongst the linked population (12,162,379), 391,233 (3.2%) had a STE in EMR data, 1,062,791 (8.7%) had a STE in claims, and 54,525 (0.4%) had a STE in both on the same day (2566 inpatient; 22,210 outpatient, and 198 other). Amongst the 54,525 individuals included, 12,853 (23.6%) had a UEA claim in EMR, 10,461 (19.2%) had a UEA claim in claims, and 9140 (16.8%) had a UEA claim in both within the 1-day window. Individuals identified as having received UEAs using claims were overall similar to those identified through EMR data, though the latter had a slightly greater proportion of Medicaid-insured patients, had slightly lower rates of chronic obstructive pulmonary disease and uncomplicated diabetes, and were slightly younger and more female-predominant (Table 1; all p < 0.05).

**Table 1:** Characteristics of included patients by receipt of ultrasound enhancing agents in claims versus electronic medical records

Baseline characteristics of individuals with UEAs identified in claims and EMR data			
Patient characteristic	Number (%) with UEA in Claims (N = 10,461)	Number (%) with UEA with in EMR (N = 12,853)	p-value
Age group (y)			**0.02**
0–17	**0 (0.0%)**	**4 (0.0%)**	
18–34	**226 (2.2%)**	**334 (2.6%)**	
35–44	**515 (4.9%)**	**669 (5.2%)**	
45–54	**1181 (11.3%)**	**1528 (11.9%)**	
55–64	**2479 (23.7%)**	**3085 (24.0%)**	
≥ 65	**6060 (57.9%)**	**7233 (56.3%)**	
Sex			**< 0.001**
Female	**4720 (45.1%)**	**5836 (45.4%)**	
Male	**5741 (54.9%)**	**6979 (54.3%)**	
Unknown	**0 (0.0%)**	**38 (0.3%)**	
Any malignancy	1676 (16.0%)	2019 (15.7%)	0.53
Metastatic malignancy	464 (4.4%)	547 (4.3%)	0.52
Cerebrovascular disease	1698 (16.2%)	1990 (15.5%)	0.12
Chronic pulmonary disease	**3169 (30.3%)**	**3726 (29.0%)**	**0.03**
Congestive heart failure	3476 (33.2%)	4150 (32.3%)	0.13
Connective tissue Diseases	406 (3.9%)	481 (3.7%)	0.61
Dementia	242 (2.3%)	309 (2.4%)	0.68
Diabetes			
With complications	2054 (19.6%)	2447 (19.0%)	0.26
Without complications	**3778 (36.1%)**	**4393 (34.2%)**	**0.002**
HIV/Aids	78 (0.7%)	112 (0.9%)	0.32
Liver disease			
Mild	1214 (11.6%)	1490 (11.6%)	0.99
Moderate or severe	128 (1.2%)	183 (1.4%)	0.21
Myocardial infarction	1565 (15.0%)	1940 (15.1%)	0.79
Paraplegia/hemiplegia	167 (1.6%)	232 (1.8%)	0.24
Peptic ulcer disease	207 (2.0%)	245 (1.9%)	0.73
Peripheral arterial disease	2368 (22.6%)	2825 (22.0%)	0.24
Renal disease	1872 (17.9%)	2253 (17.5%)	0.48
COVID-19	526 (5.0%)	671 (5.2%)	0.53

*AIDS* acquired immune deficiency syndrome, *COVID-19* coronavirus disease 2019, *EMR* electronic medical record, *HIV* human immunodeficiency virus, *N* number of individuals, *UEA* ultrasound enhancing agents, *y* year. p-values significant at a p < 0.05 level are bolded

Shown are the characteristics of included patients by whether they were identified as having received ultrasound enhancing agents (UEAs) through billing claims or electronic medical record (EMR) data within a 1-day window. P-values for Pearson’s chi-squared tests to evaluate pairwise difference across groups are shown

### Performance of claims

**Fig. 1:**
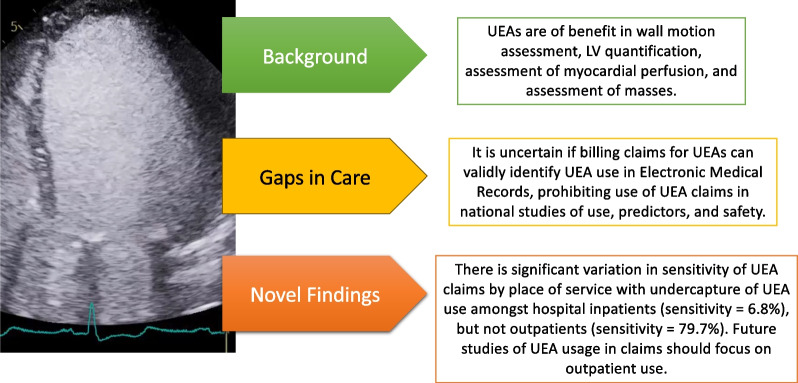
conceptual overview of study and main findings. Shown is a conceptual overview of the study background and main findings. *UEA* ultrasound enhancing agents

Overall performance of claims for detection of UEA in EMR data was high (sensitivity 71.1%, specificity 96.8%) but sensitivity was substantially lower amongst inpatients (sensitivity 6.8%, specificity 99.9%) than outpatients (sensitivity 79.7%, specificity 99.9%) (Table 2). Broadening the matching window from 1-day to 2-days (Additional file 1: Table S3), 3-days (Additional file 1: Table S4), or removing the window altogether (Additional file 1: Table S5) did not substantially change the observed results (Fig. 1).

**Table 2:** Performance of claims to identify UEA use amongst individuals with a STE in linked EMR and claims data within A 1-day window

Performance of claims to identify UEA use within a 1-day window by place of service									
		UEA in EMR Data (N)			Sensitivity (95% CI)	Specificity (95% CI)	Accuracy (95% CI)	PPV (95% CI)	NPV (95% CI)
		Yes	No	Total					
All points of service									
UEA in Claims data (N)	Yes	9140	1321	10,461	71.1% (70.3%–71.9%)	96.8% (96.7%–97.0%)	90.8% (90.5%–91.0%)	87.4% (86.8%–88.0%)	91.6% (91.4%–91.8%)
	No	3713	40,351	44,064					
	Total	12,853	41,672	54,525					
Inpatients									
UEA in claims data (N)	Yes	60	1	61	6.8% (5.3%–8.7%)	99.9% (99.7%–100.0%)	68.1% (66.3%–69.9%)	98.4% (89.3%–99.8%)	67.4% (67.0%–67.8%)
	No	817	1688	2505					
	Total	877	1689	2566					
Outpatients									
UEA in claims data (N)	Yes	3748	20	3768	79.7% (78.5%–80.8%)	99.9% (99.8%–99.9%)	95.6% (95.3%–95.9%)	99.5% (99.2%–99.7%)	94.8% (94.5%–95.1%)
	No	957	17,485	18,442					
	Total	4705	17,505	22,210					

*CI* confidence interval, *EMR* electronic medical record, *N* number of individuals, *NPV* negative predictive value, *PPV* positive predictive value, *UEA* ultrasound enhancing agents

Shown are the number of individuals with UEA receipt in electronic medical record and claims data as well as the sensitivity, specificity, accuracy, positive predictive value and negative predictive value of claims to identify UEA receipt in linked EMR and claims data. Results are stratified by point of service for the index echocardiogram, either during an inpatient hospitalization (inpatients) or in an outpatient facility or clinic (outpatients). Only UEA claims falling within 1-day of the date of service for the index echocardiogram are considered

## Discussion

In this large retrospective cohort analysis of linked EMR and claims data, while the overall accuracy of claims to identify UEA use was high, there was substantial under-capture of UEA use by claims amongst inpatients. These findings suggest that claims, while specific for UEA use, may be poorly sensitive to detect UEA use amongst inpatients. Reasons for this difference are unclear, but should motivate an improved coding system for capturing the use of this important echocardiographic adjunct.

UEAs are frequently used in echocardiography to improve left ventricular opacification and endocardial border resolution, as well as for evaluation of myocardial perfusion, evaluation of left ventricular masses, and quantification of left ventricular function [1, 9]. While UEAs have shown to be safe [10, 11] and cost-effective [3] through the reduction in downstream procedures, in single center studies, use in transthoracic echocardiography lags behind that of suboptimal image quality [12]. Moreover, gender disparities exist in receipt of UEAs, not accounted for by known clinical risk factors or image quality, with females receiving lower rates of UEAs in multiple studies [12, 13]. In this setting, while administrative claims represent an attractive tool for studying sources of variation in use of UEAs, claims have not previously been validated to identify UEA receipt, despite the prior use of claims to study UEA use in safety studies [10, 11].

In the current study, using a large all-payor database of linked EMR and claims data, UEA claims were specific for identification of UEA receipt, using EMR data as the gold standard. However, the sensitivity of claims to identify UEA receipt was substantially lower amongst inpatients (6.8%) than outpatients (79.7%). Thus, prior studies using claims to identify use amongst inpatients, including seminal safety studies [10, 11], may drastically underestimate rates of UEA utilization. As claims retain their specificity for UEA use amongst inpatients, it is unlikely that the conclusions of these studies with regard to safety of these agents are invalid. However, the rates of UEA use estimated in these studies may be substantially lower than actual practice. Reasons for this under-capture of UEA use amongst inpatients are unclear at present. However, it is possible that since the extra cost of a UEA study may be credited against a hospital’s revenue for a diagnosis-related group reimbursement, there is an incentive for hospitals to under-report use amongst inpatients. The same incentive does not exist for outpatients, perhaps explaining this differential. As such, further efforts are necessary to improve coding and capture of UEA use amongst inpatients to better understand the magnitude and impact of UEA use in national datasets. Overall, these findings suggest that UEA claims can validly substitute as an indicator of UEA administration amongst outpatients, but not inpatients, suggesting they should not be used to understand the downstream consequences of UEA use in this population. Future analyses of UEA claims data derived from the ambulatory setting may yield important insights into the use, variation, predictors of use, and downstream health benefits or safety signals from receipt of these agents.

While utilizing a large and generalizable dataset, there are several limitations of this study to consider. First, those with linked EMR and claims data in this dataset may differ than those without in ways that could influence the observed results. Second, there may be important differences in accuracy of claims for stress and transesophageal echocardiography that were not within the scope of the current study. Third, it is possible that inaccuracies in reporting of UEAs in EMRs may result in EMRs being an imperfect gold standard. Fourth, there may be differences in the accuracy of claims over time that are not captured, given the narrow focus on contemporary claims (2019–2022). Fifth, the association of UEA receipt with downstream health outcomes (including adverse events) and overall resource utilization as well as the presence of sex disparities [12, 14] in receipt of UEAs is of interest, but was beyond the scope of the current manuscript to address. Sixth, reliable information on race/ethnicity and body mass index was not available and thus not included in the current study.

## Conclusions

In this large retrospective cohort analysis of linked EMR and claims data, while the overall accuracy of claims to identify UEA use was high, there was substantial undercapture of UEA use by claims amongst inpatients. These findings suggest that claims, while specific for UEA use, may be poorly sensitive to detect UEA use amongst inpatients.

### Supplementary Information

**Additional file 1: Table S1.** Current Procedural Terminology Codes Used to Define Transthoracic or Stress Echocardiography Use. **Table S2.** Codes Used to Define Receipt of Ultrasound Enhancing Agents. **Table S3.** Performance of Claims to Identify UEA Use Amongst Individuals with a STE in Linked EMR and Claims Data within a 2-day Window. **Table S4.** Performance of Claims to Identify UEA Use Amongst Individuals with a STE in Linked EMR and Claims Data within a 3-day Window. **Table S5.** Performance of Claims to Identify UEA Use Amongst Individuals with a STE in Linked EMR and Claims Data Without Application of a Date Window.

## Data Availability

The data supporting this study are not available for review due to prior data use agreements.
