# Supplementary material for: Validation of administrative claims to identify ultrasound enhancing agent use
Source: Echo Res Pract. 2024 Feb 7;11:3. doi: 10.1186/s44156-023-00038-5 (PMC10848552; doi:10.1186/s44156-023-00038-5)
Supplement: Supplementary file 1 — Additional file 1: Table S1. Current Procedural Terminology Codes Used to Define Transthoracic or Stress Echocardiography Use. Table S2. Codes Used to Define Receipt of Ultrasound Enhancing Agents. Table S3. Performance of Claims to Identify UEA Use Amongst Individuals with a STE in Linked EMR and Claims Data within a 2-day Window. Table S4. Performance of Claims to Identify UEA Use Amongst Individuals with a STE in Linked EMR and Claims Data within a 3-day Window. Table S5. Performance of Claims to Identify UEA Use Amongst Individuals with a STE in Linked EMR and Claims Data Without Application of a Date Window. [file 44156_2023_38_MOESM1_ESM.pdf]

Additional File

Table S1: Current Procedural Terminology Codes Used to Define Transthoracic or Stress Echocardiography Use

CODE	DESCRIPTION	CATEGORY
93303	Transthoracic echocardiography for congenital cardiac anomalies; complete	TTE
93304	Transthoracic echocardiography for congenital cardiac anomalies; follow-up or limited study	TTE
93306	Echocardiography, transthoracic, real-time with image documentation (2D), includes M-mode recording, when performed, complete, with spectral Doppler echocardiography, and with color flow Doppler echocardiography	TTE
93307	Echocardiography, transthoracic, real-time with image documentation (2D), includes M-mode recording, when performed, complete, without spectral or color Doppler echocardiography	TTE
93308	Echocardiography, transthoracic, real-time with image documentation (2D), includes M-mode recording, when performed, follow-up or limited study	TTE
93320	Doppler echocardiography, pulsed wave and/or continuous wave with spectral display (List separately in addition to codes for echocardiographic imaging); complete	TTE
93321	Doppler echocardiography, pulsed wave and/or continuous wave with spectral display (List separately in addition to codes for echocardiographic imaging); follow-up or limited study (List separately in addition to codes for echocardiographic imaging)	TTE

93325	Doppler echocardiography color flow velocity mapping (List separately in addition to codes for echocardiography)	TTE
93350	Echocardiography, transthoracic, real-time with image documentation (2D), includes M-mode recording, when performed, during rest and cardiovascular stress test using treadmill, bicycle exercise and/or pharmacologically induced stress, with interpretation and report;	Stress
93351	Echocardiography, transthoracic, real-time with image documentation (2D), includes M-mode recording, when performed, during rest and cardiovascular stress test using treadmill, bicycle exercise and/or	Stress

Legend: The code column specifies the Current Procedural Terminology (CPT) code used to define the service received. The middle column specifies the description of this service. The category column specifies whether this code represents a claim for transthoracic echocardiography (TTE) or stress echocardiography (Stress).

Table S2: Codes Used to Define Receipt of Ultrasound Enhancing Agents

CODE	DESCRIPTION	CATEGORY
C8921	Add-on Code; Transthoracic echocardiography with contrast for congenital cardiac anomalies; complete	HCPCS/CPT
C8928	Add-on Code; Transthoracic echocardiography with contrast, or without contrast followed by with contrast, real-time with image documentation (2D), includes M-mode recording, during rest and cardiovascular stress test using treadmill, bicycle exercise and/or pharmacologically induced stress, with interpretation and report	HCPCS/CPT
C8929	Add-on Code; Transthoracic echocardiography with contrast, or without contrast followed by with contrast, real-time with image documentation (2D), includes M-mode recording, when performed, complete with spectral doppler echocardiography, and with color flow doppler echocardiography	HCPCS/CPT
C8930	Add-on Code; Transthoracic echocardiography with contrast, or without contrast followed by with contrast, real-time with image documentation (2D), includes M-mode recording, when performed during rest and cardiovascular stress test using treadmill, bicycle exercise and/or pharmacologically induced stress, with interpretation and report; including performance of continous electrocardiographic monitoring, with physician supervision	HCPCS/CPT
C8923	Add-on Code; Transthoracic echocardiography with contrast, real-time with image documentation (2D) with or without M-mode recording; complete	HCPCS/CPT
C8922	Add-on Code; Transthoracic echocardiography with contrast for congenital cardiac anomalies; follow-up or limited study	HCPCS/CPT
C8924	Add-on Code; Transthoracic echocardiography with contrast, real-time with image documentation (2D) with or without M-mode recording; follow-up or limited study	HCPCS/CPT

Q9957	INJECTION, PERFLUTREN LIPID MICROSPHERES	HCPCS/CPT
Q9950	INJECTION, SULFUR HEXAFLUORIDE LIPID MICROSPHERE	HCPCS/CPT
Q9956	INJECTION, OCTAFLUOROPROPANE MICROSPHERES	HCPCS/CPT
11994001104	PERFLUTREN INJECTION, SUSPENSION INTRAVENOUS	NDC
11994001116	PERFLUTREN INJECTION, SUSPENSION INTRAVENOUS	NDC
11994001720	PERFLUTREN INJECTION, SUSPENSION INTRAVENOUS	NDC
00270709707	SULFUR HEXAFLUORIDE INJECTION, POWDER, LYOPHILIZED, FOR SUSPENSION INTRAVENOUS; INTRAVESICAL	NDC
00270709708	SULFUR HEXAFLUORIDE INJECTION, POWDER, LYOPHILIZED, FOR SUSPENSION INTRAVENOUS; INTRAVESICAL	NDC
00270709973	SULFUR HEXAFLUORIDE KIT	NDC
00270709916	SULFUR HEXAFLUORIDE KIT	NDC
00407270703	HUMAN ALBUMIN MICROSPHERES AND PERFLUTREN INJECTION, SOLUTION INTRAVENOUS	NDC
00407270718	HUMAN ALBUMIN MICROSPHERES AND PERFLUTREN INJECTION, SOLUTION INTRAVENOUS	NDC

Legend: The code column specifies the particular code used to define UEA receipt. The middle column specifies the description of this code. The category column specifies whether this code represents Current Procedural Terminology (CPT) / Healthcare Common Procedure Coding System (HCPCS) code or a National Drug Code (NDC).

Table S3: Performance of Claims to Identify UEA Use Amongst Individuals with a STE in Linked EMR and Claims Data within a 2-day Window

**All Points of Service**									
		UEA in EMR Data (n)			Sensitivity (95% CI)	Specificity (95% CI)	Accuracy (95% CI)	PPV (95% CI)	NPV (95% CI)
		Y	N	Total	70.9% (70.1%-71.6%)	96.8% (96.6%-96.9%)	90.7% (90.4%-90.9%)	87.0% (86.4%-87.6%)	91.5% (91.3%-91.7%)
UEA in Claims Data (n)	Y	9,151	1,369	10,520					
	N	3,765	40,710	44,475					
	Total	12,916	42,079	54,995					
**Inpatients**									
		UEA in EMR Data (n)			Sensitivity (95% CI)	Specificity (95% CI)	Accuracy (95% CI)	PPV (95% CI)	NPV (95% CI)
		Y	N	Total	6.7% (5.1%-8.5%)	99.9% (99.7%-100.0%)	68.1% (66.3%-69.9%)	98.4% (89.3%-99.8%)	67.4% (67.0%-67.7%)
UEA in Claims Data (n)	Y	60	1	61					
	N	839	1,731	2,570					
	Total	899	1,732	2,631					
**Outpatients**									
		UEA in EMR Data (n)			Sensitivity (95% CI)	Specificity (95% CI)	Accuracy (95% CI)	PPV (95% CI)	NPV (95% CI)
		Y	N	Total	79.5% (78.3%-80.6%)	99.9% (99.8%-99.9%)	95.6% (95.3%-95.8%)	99.4% (99.1%-99.6%)	94.8% (94.5%-95.0%)
UEA in Claims Data (n)	Y	3,749	22	3,771					
	N	967	17,497	18,464					
	Total	4,716	17,519	22,235					

Legend: Shown are the number of individuals (n) with ultrasound enhancing agent (UEA) receipt in electronic medical record (EMR) and claims data as well as the sensitivity, specificity, accuracy, positive predictive value (PPV), and negative predictive value (NPV) of claims to identify UEA receipt in linked EMR and claims data. Results are stratified by point of service for the index echocardiogram, either during an inpatient hospitalization (inpatients) or in an outpatient facility or clinic (outpatients). Only UEA claims falling within 2-days of the date of service for the index echocardiogram are considered. CI = confidence interval, STE = stress or transthoracic echocardiography, Y = yes, N = no.

Table S4: Performance of Claims to Identify UEA Use Amongst Individuals with a STE in Linked EMR and Claims Data within a 3-day Window

**All Points of Service**									
		UEA in EMR Data (n)			Sensitivity (95% CI)	Specificity (95% CI)	Accuracy (95% CI)	PPV (95% CI)	NPV (95% CI)
		Y	N	Total	70.6% (69.9%-71.4%)	96.7% (96.5%-96.9%)	90.6% (90.4%-90.8%)	86.7% (86.1%-87.3%)	91.5% (91.3%-91.7%)
UEA in Claims Data (n)	Y	9,160	1,400	10,560					
	N	3,807	41,014	44,821					
	Total	12,967	42,414	55,381					
**Inpatients**									
		UEA in EMR Data (n)			Sensitivity (95% CI)	Specificity (95% CI)	Accuracy (95% CI)	PPV (95% CI)	NPV (95% CI)
		Y	N	Total	6.5% (5.0%-8.3%)	99.9% (99.7%-100.0%)	68.3% (66.5%-70.0%)	98.4% (89.3%-99.8%)	67.6% (67.2%-67.9%)
UEA in Claims Data (n)	Y	60	1	61					
	N	858	1,787	2,645					
	Total	918	1,788	2,706					
**Outpatients**									
		UEA in EMR Data (n)			Sensitivity (95% CI)	Specificity (95% CI)	Accuracy (95% CI)	PPV (95% CI)	NPV (95% CI)
		Y	N	Total	79.5% (78.3%-80.6%)	99.9% (99.8%-99.9%)	95.5% (95.3%-95.8%)	99.4% (99.1%-99.6%)	94.8% (94.5%-95.0%)
UEA in Claims Data (n)	Y	3,750	24	3,774					
	N	969	17,513	18,482					
	Total	4,719	17,537	22,256					

Legend: Shown are the number of individuals (n) with ultrasound enhancing agent (UEA) receipt in electronic medical record (EMR) and claims data as well as the sensitivity, specificity, accuracy, positive predictive value (PPV), and negative predictive value (NPV) of claims to

identify UEA receipt in linked EMR and claims data. Results are stratified by point of service for the index echocardiogram, either during an inpatient hospitalization (inpatients) or in an outpatient facility or clinic (outpatients). Only UEA claims falling within 3-days of the date of service for the index echocardiogram are considered. CI = confidence interval, STE = stress or transthoracic echocardiography, Y = yes, N = no.

Table S5: Performance of Claims to Identify UEA Use Amongst Individuals with a STE in Linked EMR and Claims Data Without Application of a Date Window

		UEA in EMR Data (n)			Sensitivity (95% CI)	Specificity (95% CI)	Accuracy (95% CI)	PPV (95% CI)	NPV (95% CI)
**All Points of Service**									
		Y	N	Total					
UEA in Claims Data (n)	Y	10,118	3,397	13,515	48.3% (47.6% - 49.0%)	94.5% (94.3% - 94.6%)	82.7% (82.4% - 83.0%)	74.9% (74.2% - 75.5%)	84.2% (84.1% - 84.4%)
	N	10,841	57,888	68,729					
	Total	20,959	61,285	82,244					
**Inpatients**									
		Y	N	Total					
UEA in Claims Data (n)	Y	73	23	96	3.7% (2.9% - 4.7%)	99.4% (99.2% - 99.6%)	68.3% (67.1% - 69.4%)	76.0% (66.6% - 83.5%)	68.1% (67.9% - 68.3%)
	N	1,892	4,044	5,936					
	Total	1,965	4,067	6,032					
**Outpatients**									
		Y	N	Total					
UEA in Claims Data (n)	Y	4,098	936	5,034	60.3% (59.2%- 61.5%)	96.1% (95.8% - 96.3%)	88.1% (87.7% - 88.5%)	81.4% (80.4%- 82.4%)	89.4% (89.2% - 89.7%)
	N	2,694	22,800	25,494					
	Total	6,792	23,736	30,528					

Legend: Shown are the number of individuals (n) with ultrasound enhancing agent (UEA) receipt in electronic medical record (EMR) and claims data as well as the sensitivity, specificity, accuracy, positive predictive value (PPV), and negative predictive value (NPV) of claims to identify UEA receipt in linked EMR and claims data. Results are stratified by point of service for the index echocardiogram, either during an inpatient hospitalization (inpatients) or in an outpatient facility or clinic (outpatients). Any UEA claim is considered a possible match, disregarding the date of service for the index echocardiogram. CI = confidence interval, STE = stress or transthoracic echocardiography, Y = yes, N = no.
